# Secondary school students’ and peer educators’ perceptions of adolescent education in rural Tanzania: a qualitative study

**DOI:** 10.1186/s12978-022-01418-6

**Published:** 2022-05-02

**Authors:** Keiko Ito, Frida E. Madeni, Yoko Shimpuku

**Affiliations:** 1 Kyoto University Hospital, 53 Shogoin-kawaharacho, Sakyo-ku, Kyoto, 606-8507 Japan; 2 Korogwe Town Council, Korogwe, P. O. Box 615, Tanga, Tanzania; 3 Graduate School of Biomedical and Health Sciences, Hiroshima University, 1-2-3 Kasumi, Minami-ku, Hiroshima, 734-8551 Japan

**Keywords:** Adolescent education, Peer education, Focus group discussion

## Abstract

**Background:**

In many African countries, cultural norms and values hinder conversations about sexuality among adolescents and their parents. Currently, there are no sex education classes in the curriculum at schools in Tanzania. Even when sex education is provided, the content is often abstinence-oriented, and there is a lack of in-depth instruction and exploration on the topic. To help overcome this, peer education is encouraged. After implementing peer-based adolescent education via a non-profit organization, this study aims to (1) identify students’ and peer educators’ perceptions of adolescent education and (2) identify the changes that occur as a result of adolescent education with peer educators.

**Methods:**

This was a qualitative descriptive study using focus group discussions (FGDs). Secondary school students, including peer educators as well as students who received adolescent education, were asked about their perception of peer-based adolescent education. The FGDs were conducted in Swahili with the support of local collaborators. Data were transcribed and translated into English and Japanese. Content analysis was conducted to merge the categories and subcategories.

**Results:**

A total of 92 students (57 girls and 35 boys) were included from three urban and three rural secondary schools where peer education was being implemented. Six FGDs were conducted for girls and four for boys, for a total of 10 FGDs. The students had both positive and negative perceptions of peer-based adolescent education. Both the peer educators and the other students felt that they gained more confidence through the process, based on the conversations they had and the trusting relationship that formed as a result. The peer educators were also successful in eliciting behavioral changes, and the students shared their sex-related knowledge with other peers as well.

**Conclusion:**

The peer education process helped students gain confidence in teaching their peers and elicit behavioral changes. Adult supervision for peer educators is suggested.

**Supplementary Information:**

The online version contains supplementary material available at 10.1186/s12978-022-01418-6.

## Plain Language Summary

Peer education, such as sharing correct knowledge, skills, and behaviors, is encouraged for maintaining a healthy lifestyle in later life. The non-profit organization Class for Everyone conducted adolescent education in collaboration with a local NGO, the New Rural Children Foundation, to prevent unwanted pregnancy and social isolation among adolescent girls. In secondary schools, the NGO members provided adolescent education led by peer educators.

In this study, we conducted focus group discussions to understand how peer educators and other secondary school students perceived peer-based adolescent education. The study included 92 students (57 girls and 35 boys) from three urban and three rural secondary schools where peer education was being implemented. We used content analysis to merge the categories and subcategories.

We found that students had both positive and negative perceptions about peer-based adolescent education. The participants felt that they gained more confidence through the peer education process. The peer educators were also successful in eliciting behavioral changes. Moreover, the students receiving peer education shared their sex-related knowledge with other peers as well.

In conclusion, the peer education process helped students gain confidence in teaching their peers and elicit behavioral change. Adult supervision for peer educators is suggested.

**Supplementary Information:**

The online version contains supplementary material available at 10.1186/s12978-022-01418-6.

## Background

The World Health Organization [1] reports that pregnant girls under 15 years of age are at high risk of maternal mortality due to complications such as prolonged labor, obstructed labor, postpartum bleeding, and unsafe abortions. According to the Demographic Health Survey [2] in Tanzania, among individuals aged 18–24 years, more than 60% of women and 51% of men experience their first sexual intercourse before the age of 18 years. Further, 56.7% of girls under 19 years of age have experienced childbirth. Because Tanzanian girls are forced to drop out of school if they are pregnant, many students choose abortion to avoid this consequence [3]. In Tanzania, abortion is illegal, and there are only a limited number of facilities where abortions can be safely performed. Therefore, abortions are sometimes performed in non-medical facilities, mainly in rural areas, using traditional methods such as the use of strong medicinal herbs and insertion of the cassava root into the vagina [3].

Being unable to continue with their studies has a significant impact on the lives of female students. They may also be abandoned by their families due to social prejudice [4], which puts them at a great economic and social disadvantage. The percentage of students who dropped out of school due to pregnancy was 15% in 2016 [2], amounting to more than 8000 students per year [5]. Female students may also experience psychological distress after delivery due to stillbirth and birth fistula [6].

Currently, there are no sex education classes in Tanzania. However, life skills classes are often taught by teachers with no special training [7]. In addition, the content is often abstinence-only sex education and has insufficient in-depth instructions [8]. The lack of knowledge among teachers and their lack of confidence in teaching this topic is another reason why students are not encouraged to understand this topic [9].

In many African countries, cultural norms and values make it taboo to discuss sexuality with parents at home, and parents themselves are reluctant to discuss sexuality with their children because they feel that they do not have adequate information [9–12]. Even when parents discuss sex with their children at home, it is often one-sided and prohibitive rather than knowledge-providing [13, 14]. Children are afraid that their parents will suspect them of doing something “wrong” when they ask questions about sex [9]. As they are unable to discuss their sexual concerns at home, adolescents tend to gain knowledge about the topic by talking to their friends [15, 16].

As a solution to this concern, peer education, such as sharing accurate knowledge, skills, and behaviors, is encouraged [17]. Adolescents spend a great deal of time with peers belonging to the same age and generation. They influence each other in positive and negative ways. Peers are also a source of information about sexuality [18, 19]. Somefun and Odilmegwu [20] stated that knowledge and habits acquired during adolescence provide the foundation for maintaining a healthy lifestyle in later life. To disseminate accurate knowledge, peer education can be more interesting and thought-provoking because it is taught by close and trusted peers [21]. Furthermore, it is effective in developing norms for future behavior [22]. Previous studies have reported that peer education is an educational method that helps students think about sex in a positive, constructive, and proactive way [23]. In addition, the concept of adolescent education through peer education is based not only on sharing knowledge but also on accepting others and listening to their thoughts, which contributes to “kindness” in caring for others and in forming relationships [24]. It may help students become aware of the changes in their own bodies and take care of not only themselves but also their friends of both the same and opposite sex.

According to Share-Net Netherland [7], students who received peer education interventions were more likely to be aware of their own bodily changes than those who did not. The authors also reported that students who received peer education interventions not only increased their knowledge about sexuality but also their problem-solving skills and self-esteem, as compared to students in the non-intervention group. To the best of our knowledge, two studies on peer education have been implemented in African countries, including Tanzania [25, 26]. However, they only discussed the effects of the intervention; there was no inclusion of the perceptions of adolescents. We were unable to find any previous research examining adolescents’ perceptions of peer education and the changes that occurred in the students as a result of peer education. Therefore, the purpose of this study is twofold: (1) to identify students’ and peer educators’ perceptions of adolescent education and (2) to identify the changes that occurred as a result of adolescent education with peer educators.

## Methods

### Research design

This qualitative descriptive study uses focus group discussions (FGDs), which aim to obtain information from a variety of perspectives that cannot be obtained through individual interviews by bringing together people with specific experiences and thoughts [27]. In this study, the FGD method was chosen with the aim of eliciting students’ experiences and thoughts more effectively through student interaction, as students can motivate each other to speak up in regard to both sides: students and peer educators.

### Setting

The non-profit organization Class for Everyone conducted adolescent education as a Japan International Cooperation Agency (JICA) Partnership Program between April 2017 and September 2019 in collaboration with a local NGO, the New Rural Children Foundation, to prevent unwanted pregnancy and social isolation among adolescent girls. For this project, adolescent education was defined as not only teaching sexuality or the prevention of unwanted pregnancy but also providing students a space in which to consider relationships with others, decision-making for their life plan, and values necessary to live in the community as a member. This project was conducted in primary and secondary schools in the Korogwe District, Tanga Region, which was nearly 300 km away from Dar es Salaam, the biggest city in Tanzania. There were 43 primary and 15 secondary schools in the three wards of the district, all of which were included in the project. In secondary schools only, peer education was introduced by the NGO members. Therefore, this study included only secondary schools. The authors communicated and collaborated with the project team and collected the data after the project was completed.

### Participants

The study participants included peer educators and students receiving peer education in grades 1, 2, and 3 (approximately 13–18 years old) in secondary schools. We included participants who understood the purpose of the study and agreed to participate and who were able to read and write in Swahili. Peer educators were recommended by the teachers at each school, and 3–9 peer educators were selected for each grade, depending on the size of the school. In this study, students who received adolescent education by peer educators were invited to participate to satisfy the data triangulation by collecting opinions and thoughts from different viewpoints [28].

An FGD is conducted in an informal group with a small number of participants (usually about 6–12) to discuss a selected topic [29]. In this study, there were approximately 10 male and female peer educators in each school. Around 10 research participants were also recruited among students who had received adolescent education from peer educators. We adjusted the number of participants so that the total number of participants, together with the peer educators, was around 10. The demographic information of the research participants who agreed to participate in the study was checked to ensure that there were no extreme gender differences.

We set the exclusion criteria as students who had experienced or almost experienced sexual abuse or forced sexual intercourse and who felt uncomfortable with or may be traumatized by questions about adolescent education. During the informed consent process, we asked if any students were uncomfortable discussing adolescent education; students who indicated that they were uncomfortable would not be forced to participate in the study. However, because no students indicated as such, none were excluded.

### Data collection

Data were collected in October 2019. Interviews were conducted with the local NGO staff, who were also the research collaborators for this study. Before starting recruitment and data collection, we exchanged opinions with the local NGO staff on the appropriateness of the questions in the interview guide. We visited six secondary schools where peer education for adolescents was being implemented, met with the principal or vice principal, explained the contents of the survey, and obtained approval to discuss it with the students. We convened the students who acted as peer educators, explained the purpose and methods of the study in writing and orally, and obtained their approval to participate in the study. We asked the peer educators to bring their friends who had received adolescent education from peer educators. We explained the purpose and methods of the study in writing and orally to these students as well and asked them to participate in the study.

From the perspective of creating an environment in which students could express themselves comfortably, as the content of the study included sex-related topics, the research participants were separated by gender. After obtaining consent from the research participants, the FGDs were recorded using voice recorders. FGDs were conducted with two Tanzanian collaborators (a male in his 30s who was a social welfare worker and sociology degree holder and a male in his 30s who was local NGO staff). FGDs were conducted in vacant classrooms or in quiet places, such as the courtyard. At the end of the FGDs, the participants were asked to fill out a face sheet with basic information about themselves (age, gender, grade, religion, tribe, number of siblings, and menstruation and ejaculation). The interview guide was attached as a Additional file 1.

During the FGDs, the first author asked questions according to the interview guide and wrote down the main points in the field notes, adding questions as required while listening to the students’ comments. As the FGDs were conducted in Swahili, the first author translated the Swahili into English, and the transcript of the recorded data was checked and revised with the research collaborator to ensure the accuracy of interpretation. After that, the first author further translated the transcript from English to Japanese so that the analysis was conducted in three languages, and all authors could check the categorization in their native language.

### Data analysis

The transcripts from the FGDs were analyzed using the content analysis method, which is a frequently used method for qualitative data analysis, especially for qualitative descriptive research [30, 31]. The content analysis method was suitable for this study because it aims to deepen the interpretation of the obtained data through repeated reviews and to provide a rich and direct analysis based on experiences and facts [32]. To allow reference to the original-language responses at any time, a verbatim transcript was created with Swahili, English, and Japanese, side by side. After the verbatim transcription was completed, the first and third authors, who regularly conducted research in Swahili, reviewed and coded all of the statements from the research participants, searched for similarities, commonalities, and patterns, and categorized them.

### Ethical considerations

The research participants were given written and verbal explanations of the content of the study. The research was conducted following ethical principles: participation was voluntary, participants had the freedom to withdraw from participation during the study, and their personal information was protected. Consent was obtained via their signatures. Regarding the background information of the research participants, they were not required to write their names or their school’s names. This study was approved by the Kyoto University Graduate School of Medicine Ethics Committee (R1911) and the National Institute of Medical Research of Tanzania (NIMR/HQ/R.8a/Vol/IX/988).

## Results

### Background of the participants

The study participants consisted of 92 students (57 females and 35 males) who were peer educators or who had received adolescent education from peer educators in three urban and three rural secondary schools where peer education was being implemented. Six FGDs were conducted for girls and four for boys, for a total of 10 FGDs. Details of the study participants are presented in Table 1.

**Table 1:** Background of the participants

	School	Gender	Number of participantss by role		Average age	FGD time
			Peer educators	Students who received adolescent education by peer educators		
Town	A	Boys	14	0	15.5(14–17)	45:45
		Girls	12	0	15.0(14–16)	1:02:09
	B	Boys	5	4	15.4(14–18)	1:12:25
		Girls	13	0	14.6(13–16)	1:28:00
	C	Girls	7	4	15.9(15–17)	40;49
Rural	D	Boys	4	4	16.3(14–18)	1:28:00
		Girls	4	1	15.6(15–16)	1:33:30
	E	Boys	3	3	15.8(15–17)	44:11
		Girls	4	4	16.3(15–18)	34:50
	F	Girls	6	0	15.5(14–18)	50:04

#### Students' perceptions of adolescent education with peer educators

The participants’ statements included both positive and negative perceptions. Among them, two categories and five subcategories were generated for positive perceptions, and two categories and four subcategories were generated for the negative perceptions (Table 2).

1. Positive perceptions

**Table 2:** Students' perceptions of adolescent education with peer educators

Positive perceptions	Benefits of student-to-student relationships	A close relationship can aid in teaching and learning
		A sense of trust based on established relationships
		Increased motivation through friendship
	Ripple effects of encouragement and motivation to solve problems	Peer educators and students motivating each other
		Desire to implement the education outside of school (in the community)
Negative perceptions	Difficulties in gaining trust as a teacher	Being insulted and disrespected while teaching
	Difficulties in teaching caused by students	Difficulties in changing behaviors due to culture and customs
		Difficulties in changing behaviors due to the living environment
		Difficulties in teaching older friends

#### Benefits of relationships among students

*A close relationship can aid in teaching and learning*

The peer educators and the students taught by peer educators were friends. Both felt that they could learn, teach, and understand each other regarding a sensitive topic such as sexuality because of their close relationship. At the same time, they were happy to have their friends listen to their concerns and questions about sex, and they were motivated to promote their understanding of their daily lives and share it with other friends.

“It's a good feeling because we are all on the same page and can teach each other. It made me realize that if I have a friend who is doing something undesirable, it is good to bring them back to the right place.” (Rural School E, peer educator, male student)

“When they first came to teach us, we felt good because they are our friends, they are always with us, they listen to us, and they understand us. Therefore, we understood what they were teaching us, and we tried to follow what they taught us.” (Rural School D, male student)

“The peer educators listened to me very well. The peer educators taught me to think about how I felt about sexual issues.” (Rural School E, female student)

#### A sense of trust based on established relationships

As the peer educators and other students already knew each other and spent a great deal of time together at school, it was easier for them to talk about sexual issues with each other. They viewed adolescent education through peer education positively. Outside of peer education, it had been difficult to teach this topic because (1) parents and teachers tended to provide the students with only general information, (2) children were afraid to ask adults questions on this topic, and (3) it was difficult to talk to outsiders such as NGO staff because they did not know when they would return.

“When students teach each other, they can talk freely about sex. However, if there are people from outside, I would feel embarrassed to talk about it and hide it. With familiar friends, we can talk freely about what we feel without hiding it.” (Urban School C, female student)

“If you have talked about sex with your parents when you were young, you can ask them questions. However, some parents did not answer correctly. Sometimes, they warn them not to do this. However, with students, everyone is on the same page, and they can ask questions freely without fear.” (Urban School B, peer educator, male student)

“We already know each other well, so it's good for us to teach each other. Teachers only teach us general things. However, with other students, we can go to our friends privately and keep teaching them until they understand well.” (Urban School A, peer educator, male student)

*Increased motivation through friendship*

We found that the students became motivated to share what they had learned with others to help them avoid encountering similar problems. It was also found that the students were influenced by the attitudes and actions of their motivated peer educators.

“Peer education in schools has had a positive impact on reducing the number of young pregnancies and helping the stubborn students who are reluctant to listen to others more understand. If they start having sex when they are still young, their performance in school will also suffer. As a teacher, I want to teach and help more people understand.” (Rural School E, peer educator, male student)

"I teach my friends and peers about the negative effects of sex and abortion because if you have an abortion, you may have problems with your reproductive organs. If you get pregnant later, you might be miscarrying. You will not be able to continue your studies, and you will have to quit school.” (Rural School E, peer educator, female student)

#### Ripple effects of encouragement and motivation to solve problems

*Peer educators and students motivating each other*

Students who had received adolescent education from peer educators helped and encouraged peer educators in their activities, which, in turn, inspired the peer educators to teach other friends.

"We teach our friends, and they understand and encourage us. This makes us want to teach others more.” (Rural School E, peer educator, female student)

"The people I taught understood better than I did and became good teachers. If I do not answer a question or make a mistake, he comes and tells me that this was wrong. It is nice to know that the friends I have taught are helping me.” (Urban School B, peer educator, male student)

“I wish I could become a teacher so that I could teach my friends.” (Rural School D, female student)

*Desire to implement the education outside of school (in the community)*

The students expressed the desire to conduct peer education outside school as well. Adolescents outside the school also wished for support from the community for teaching so that the residents knew that they were working as peer educators.

"We should bring this education to the people in our village. For example, we all want to go to the village and teach the villagers so that they can have the correct knowledge like we do.”(Urban School C, Peer Educator, Female Student)

“I think it's better not to restrict this education only to schools. I think it’s better that this education is provided not only at school. If it ends here only at school, other people won't be able to hear.” (Rural School F, peer educator, female student)

“I think we need to expand our activities so that people don't think it's only a problem in our province, but it's the same in other places. If we do this, I think the number of people who want to study will increase in Tanzania. I think we need to look more at the future and not just the present.” (Urban School B, peer educator, male student)

“We have a lot of friends in our community who have failed at their primary school exams and are not getting an education, and these friends are having adolescent pregnancies. I think if we teach ourselves outside of school, if we understand ourselves well and have the right knowledge about sex, we will rarely have adolescent pregnancies.” (Urban School A, peer educator, male student)

(2) Negative perceptions

#### Difficulties in gaining trust as a teacher

*Being insulted and disrespected while teaching*

Many peer educators experienced being insulted or belittled while teaching their friends. Some students who received adolescent education from peer educators did not trust the way in which they taught, which was sometimes in an unconfident manner. Some students initially felt disdain in regard to being taught by their peers. However, these feelings gradually transformed into a sense of trust as peer educators continued to teach without giving up.

“I was sad when I was teaching my friends. They just kept trying to avoid me or ignore me and were doing bad things.” (Rural School E, peer educator, male student)

“When I taught one person, she said, ‘Don't confuse me! Why do you need to teach me?’” (Urban School C, peer educator, female student)

"At first, I despised him. I thought he was just a normal friend and that he was here to waste my time. However, every day he kept coming back to teach, and I started to listen to him.” (Urban School B, male student)

#### Difficulties in teaching caused by students

*Difficulties in changing behaviors due to culture and customs*

Peer educators felt that adherence to the local culture and customs caused difficulties in their activities when promoting behavioral changes among their friends.

“For example, the culture of female circumcision and child marriage. As a peer educator, it is difficult to talk about these issues.” (Rural School F, peer educator, female student)

“We are told to get married at the age of 13. If I do not listen to this at home, my family will think I am a bad daughter and teach me about marriage.” (Rural School F, peer educator, female student)

"I think the tribe also has an influence. I think it is partly due to tribal influences because sometimes people think that once you have had your first menstruation, you are grown up and can live on your own. I think these things can contribute to people who have sex.” (Urban School C, peer educator, female student)

*Difficulties in changing behaviors due to the living environment*

Some of the students had sexual relations in exchange for money or daily necessities. Moreover, some students had relationships with peers from groups with reputations that included harmful behaviors. Despite having adequate knowledge about sexual relationships, the harshness of the students' home environments often made it difficult for them to change their behaviors, owing to poverty.

“Even if the teacher (peer educator) tries to teach us, the harshness of life can make it difficult to listen. If we want to implement what they teach us, we feel like where the hell we are going to get what we need for our lives?” (Rural School E, female student)

“Poverty can cause students to engage in undesirable behaviors. Adolescents look for people who can help them fulfill their needs.” (Rural School D, male student)

“In a poor family, if a girl gets married, the family can get five cows, maybe five goats. Because of poverty, parents force their children to marry to get these rewards.” (Rural School F, peer educator, female student)

*Difficulties in teaching older students*

Peer educators found it difficult to talk about sex with their older friends.

“I think if I taught someone older than me, they would despise me. If it is someone younger, it's okay. If it were a school, I would not go to teach Form 3,4 sisters (older students).” (Urban School B, peer educator, female student)

“The most difficult thing for me has been my age. It is usually my older friends who are behaving in an undesirable way. Everyone can see that they are behaving badly. However, it is hard to tell older friends that they were wrong. Even mothers or fathers may find it difficult. You will think about how to tell your friends. We can see that our friend is going in the wrong direction in life. But if you say, I am here to teach you because I have studied, your friend may also say, I studied before you.” (Urban School A, peer educator, male student)

### The changes that occurred as a result of adolescent education with peer educators

The changes resulting from adolescent peer education include changes occurring in peer educators and students (Table 3).

1. Changes in peer educators

**Table 3:** The changes that occurred as a result of adolescent education with peer educators

Changes in peer educators	I was given a role	I am now called teacher
		I felt the joy of being chosen by God
	I wanted to gain the trust of my friends	I needed to change first to teach my friends
		I realized how important it is to have confidence as a teacher
	I found it meaningful	I felt the joy of seeing my friends change because of what I taught them
		I understood that the activities I was doing were meaningful
		I have many more friends to teach, so I wanted to continue
Changes that occured in students who received adolescent education from peer educators	I gained the right knowledge	I understood more through continuous discussion
		I no longer feel anxious about secondary sexual characteristics
		I felt a sense of freedom and security in being able to talk about anything
	I wanted to do the right thing	I gained the confidence to say no
		I want to share what I was taught with other friends
	I gained control over my sexual interests and desires	I know what I should and should not do
		I learned about negative consequences
		I understand what is not necessary for me right now

**“I was given a role”**

***“I am now called teacher”***

The title “teacher” made the peer educators feel special.

"My friends call me by my name, 'Teacher B.’ Everyone knows my name, like the president.” (Urban School B, peer educator, female student)

“I was very happy to be called a teacher because I feel like I have something special when I am called a teacher even though I am a student. As a teacher, I feel joy in spending time with my students (friends) and teaching them. It makes me happy because I get to have a career like no other.” (Urban School A, peer educator, male student)

***“I felt the joy of being chosen by God”***

Some students felt that the role of peer educators was given to them by God, and this gave them joy and motivation in their activities.

“In Islam, God says that there is nothing that cannot be done. So, it is not the teacher, but God, who chose us. This is one of the things that motivates us to keep doing what we do.” (Urban School B, peer educator, female student) “I think the work of peer educators is a God-given job, and we should all work even harder.

We Africans think that we get paid for what we do, but according to me, we should think that we are doing God's work. If we can teach our friends, we will be blessed by God.” (Urban School A, peer educator, male student)

**“I wanted to gain the trust of my friends”**

***“I needed to change first to teach my friends”***

The students who were given the role were able to reflect on their own behavior. They believed that if they were doing the right thing, they could teach it to their friends. They also felt that they could not get their message across to their friends, even if they told them about their undesirable behaviors. Some students recognized the importance of letting go of their shame so that they could better communicate and teach effectively.

“Before I could go and teach my friends, I had to change myself. You cannot tell your friends that they should quit when you are doing something wrong. So, I started by making an effort to change myself. I used to like to listen to music and watch TV at night, but I got an education and now I study hard. I teach this to my friends.” (Rural School D, peer educator, female student)

“We need to let go of our embarrassment and teach because if we teach while feeling embarrassed, our friends will not understand what we want to say. To teach, we need to change our own behavior so that our friends can clearly see that we have changed. If someone who is behaving in an undesirable way goes to another friend and tells them about it, they will not listen. Let us change our own behavior first, then go to our friends and teach them.” (Urban School A, peer educator, male student)

***“I realized how important it is to have confidence as a teacher”***

Some of the students who were chosen to be peer educators were anxious at first. However, they realized that it was important to have confidence in their roles as they carried out the activities. When peer educators were confident, the students around them were more likely to listen to them, and this became the foundation for building trusting relationships.

“The benefit I got is that I believe in myself. First, you need to show that you are confident in providing sex education to your friends. Then, you need to tell your friends what not to do and what is good to do. Then, it will be easier to tell them about ways to avoid temptation.” (Rural School F, peer educator, female student)

“Peer educators encounter difficulties because they seem unsure about talking about sex, as if they are talking about it for the first time. When they are shy, it seems a little strange to the people listening. Therefore, they should teach with confidence. If you talk like a doctor, it is normal. So, I think it all comes down to confidence. They should talk about it in a way that it feels normal to everyone.” (Rural School D, male student)

**“I found it meaningful”**

***"I felt the joy of seeing my friends change because of what I taught them”***

The peer educators felt a sense of joy and pride and recognized their own abilities when they noticed that their friends had changed their behavior.

“I was happy to see that the friend we taught completely stopped being bad and started being good. Now, I think my friend is doing the right thing better than I am.” (Urban School C, peer educator, female student)

"The friends I have taught have changed and are now studying hard. Teaching as a peer educator has made me more comfortable while interacting with other people and has given me the confidence to teach others.” (Urban School B, peer educator, male student)

“The more I teach, the more morals I gain. The more I teach, the more my friends behave better than they used to.” (Urban School B, peer educator, male student)

“This education gave me confidence, and my friends believed in me because my friends who are not peer educators know that I have already received sex education and have knowledge.”(Urban School A, peer educator, female student)

***“I understood that the activities I was doing were meaningful”***

Peer educators found it meaningful when their friends changed because of their teaching, when their friends thanked them, and when their friends understood what they said and started helping them in their activities.

“I was grateful to God and felt that my activities were meaningful. This is because I have been able to lead others.” (Urban School B, peer educator, female student)

“Some of my friends have actually changed their behaviors. Some of them have come to me to thank me. That was a reward from God for me, or in other words, I was very happy.” (Urban School B, peer educator, female student)

“Some of them were acting out of morals, and some friends didn't understand themselves. But when I started to teach them, they changed until they studied harder and harder. I thank God.” (Urban School B, peer educator, male student)

***“I have many more friends to teach, so I wanted to continue”***

Although the students sometimes faced difficulties in teaching their friends, they felt confident and motivated by their friends’ behavioral changes and wanted to reach out to other friends as well.

“We have seen changes in some of the people who needed to change their behaviors. Not all of them have changed, and I want to keep working on this. I think we need to teach them more so that they understand better.” (Rural School E, peer educator, female student)

“The thing that still makes us want to continue peer education is that if none of the people we taught had changed, we would have lost motivation long ago, and we would have thought that what we were teaching was nonsense and that no one would change. However, for example, a girl who was in the wrong group in the class changed her mind that this was not the right thing to do, and she left the group and went back to where she belonged. Things like this keep us away from giving up hope and continuing to teach our friends.” (Urban School C, peer educator, female student)

(2) Changes that occurred in students who received adolescent education from peer educators.

**“I gained the right knowledge”**

***"I understood more through continuous discussion”***

A school provides an environment where students spend a great deal of time together and creates opportunities for continuous discussion among students, which, in turn, promotes their understanding.

“They're our friends, they're always with us, they listen to us, and they understand us. Therefore, we understood what they were teaching us, and we tried to follow what they taught us.” (Rural School D, male student)

“In school, you have a lot of time to discuss and give advice to your friends. Of course, you can obtain education outside of school. But at school, you can believe in yourself first, and your friends can give you advice on things that you cannot solve on your own. You can learn good ways to solve these problems.” (Urban School B, male student)

***“I no longer feel anxious about secondary sexual characteristics”***

Through adolescent education, students had the opportunity to learn about the various physical changes that occur with secondary sexual characteristics. Students mentioned that their anxiety was reduced, and they were able to understand these changes correctly with the guidance of their peer educators.

“Before sex education, I thought that my breasts would grow when I got my period. But now I know that there are different stages, and they do not happen at the same time.” (Urban School B, female student)

“I used to get scared when I saw my friends getting their periods, thinking that God would make my stomach hurt. But now I know that it is normal, and that all girls go through it when they grow up. It is not something that you can decide for yourself whether or not you are going to have your period; it's a change that happens to everyone.” (Rural School E, female student)

***“I felt a sense of freedom and security in being able to talk about anything”***

As a result of the trusting relationship, students felt free and safe to talk about anything without any restrictions, as compared to talking to their parents or teachers. We found that the students felt a sense of joy and security in knowing the truth.

“I feel happy when my friends tell me the truth. The teacher hides the things from us. I am happy when they tell us the truth.” (Urban School B, male student)

“I feel safe when I am being taught by my friends especially because what I am being taught is about sex. However, it would be completely different if someone else taught me. There may be a sense of shame. I think it's easier to understand than being taught by someone from outside.” (Urban School B, male student)

“If we teach each other, we can talk freely, but if there are people from outside, we would feel embarrassed and hide things from them. However, with close friends, we can talk freely without hiding what we feel.” (Urban School C, female student)

**"I wanted to do the right thing”**

***"I gained the confidence to say no”***

We found that receiving adolescent education from the peer educators and gaining the correct knowledge provided the students with an opportunity to reflect on their own behavior. By doing so, they gained self-confidence and the courage to say no to protect themselves.

“Sex education gave me the confidence to refuse unwanted temptation and actions. For example, I used to be too afraid to say anything, but with continued sex education, I am no longer afraid to say what I do not like.” (Urban School C, female student)

“Before I received sex education, I was a nuisance who went to many girls. However, after I received sex education, I was able to change a little. I used to not go home, but now I think that it is better to watch a movie than to go to different girls.” (Rural School E, male student)

***“I want to share what I was taught with other friends”***

We found that students who had been taught by peer educators yearned to be one themselves, and they wanted to share what they had learned with their friends.

“We passed on the sex education we had been taught to our friends. We also told them that there were many challenges for the young.” (Rural School E, female student)

“I now wish I could be a teacher so that I can teach my friends.” (Rural School D, female student),

**"I gained control over my sexual interests and desires”**

***“I know what I should and should not do”***

Students who gained the correct knowledge through peer education gained clarity on what they should do as secondary school students, and they expressed a desire to work toward their goals. It also helped them gain the courage to tell people what they are doing wrong.

“This peer education we received helped us study hard and achieve our dreams and goals. We will be able to avoid all sorts of bad temptations and move on to the next stage of our lives after finishing our schooling.” (Urban School C, female student)

“I learned that you could get pregnant even if you have only had sexual intercourse once. Therefore, we can protect ourselves. For example, even if a girl used to be too close to a male teacher, she would no longer do so. If a male teacher is about to misbehave with a student, she will have the courage to go to the school principal or student advisor.” (Urban School C, female student)

***“I learned about the negative consequences”***

Learning about the various negative consequences of sexual relationships led to feelings of fear and a desire to avoid the negative consequences. This led the students to think that it would be better not to engage in sexual relationships.

“I was scared to learn about the negative consequences of having sex while I was still young, such as abortion and sexually transmitted diseases. I also learned that if I did not have sex, I would not have those problems.” (Rural School E, male student)

“I learned about many negative effects, such as HIV/AIDS, teenage pregnancy, and death during childbirth. Even if you do not die, you can still bleed a lot. This can have a significant impact on health. Therefore, education should be continued in schools.” (Urban School B, male student)

***“I understand what is not necessary for me right now”***

The students felt that they should stop doing what was “not necessary for them” as secondary school students and do what was required of them.

“I used to think that having sexual intercourse would be much more beneficial than continuing my studies. However, when my friend came to teach me, I decided that studies are more important now, and I should quit having sex.” (Rural School E, female student)

“I think you should have a strong opinion about things. I think it is important to be aware that you are a student and that you need to be in a position to study hard rather than hoping for something you cannot do.” (Rural School D, male student)

## Discussion

### Confidence gained by peer educators and students

The results of this study showed that the participants, including both the peer educators and the students who received adolescent education, gained confidence through the process of peer education. The Joint United Nations Programme on HIV/AIDS (UNAIDS) [33] reported that peer education works not only for peer educators but also for the beneficiaries because as it follows a horizontal and participatory style of learning.

In this study, some students were initially anxious about being selected as peer educators, but as they continued with the peer education-related activities, their anxiety disappeared. Many of them said that it was important for them to have confidence in their activities as peer educators. Mason-Jones, Mathews, and Flisher [34] reported that peer educators' credibility is not earned automatically but through their experiences, their personality, and the messages that they deliver. Many of the peer educators experienced difficulties in carrying out the peer education-related activities, including being insulted and disrespected by their friends; however, they persevered. In a study by Mason-Jones et al. [26], peer educators also felt a strong sense of camaraderie and a sense of responsibility to help their friends go through school without encountering problems such as teenage pregnancy. Furthermore, in the current study, the peer educators felt happy when their friends listened to them and changed their behaviors. The feeling of being trusted by their friends may have contributed to the peer educators' increased confidence. Chikamoto [34] stated that it is essential to increase health educators’ self-efficacy to promote successful behavioral change among participants. Further, it is necessary to create an environment in which health educators can challenge themselves with confidence. She also stated that receiving praise for what has been done is necessary. For peer educators to continue their activities with confidence, creating a space where they are praised for their efforts, either by adults who are supervising them or by other peer educators, could be effective.

Regarding the students who received adolescent education, not all of them viewed the presence of peer educators positively from the beginning. Some had negative thoughts and were distrustful regarding whether the peer educators were approaching them because they thought that they had problems or whether their peers were capable of helping them at all. Sommer et al. [35] found that peer educators’ attitudes, such as the fact that they never gave up and were consistent in their efforts and that they listened to students and tried to understand their perspective, gradually led to a change in the way the students around them opened up to them. The researchers reported that there is a lack of opportunities for teaching Tanzanian boys about the physical and emotional changes occurring during puberty. As this study shows that students experienced reduced anxiety and increased confidence after receiving accurate information about secondary sexual characteristics, increasing the opportunities for students to receive this information is key in determining whether students will choose the right health behaviors later in life.

On the other hand, some students were resistant to change due to the influence of local culture and the fact that the adolescent education was being imparted by their peers [36]. In this study, the peer educators persevered and continued to interact with their friends in a positive and accepting manner. The fact that encouragement by the peer educators helped students gain the confidence to say “no” to things they did not like is particularly noteworthy. This is because in many sub-Saharan African countries, it is not uncommon for students to have sexual intercourse even if they do not wish to in order to gain money, daily necessities, and other necessities for themselves in exchange because of poverty and other family circumstances, as described by the students in this study [3, 4, 6, 18]. In addition, male-dominated social structures are still in place, especially in rural areas where people are reportedly forced to have sex with men and are subjected to sexual violence if they say no [3]. A report by UNICEF [37] stated that adolescents misunderstand what “consent” means and are unaware of the fact that they can say “no” to sexual engagement as well. Thus, it is noteworthy that the students gained confidence in their ability to say “no” to unwanted sexual behaviors. On the other hand, given the culture and background of the area, there is a risk of violence as well as an emotional and financial disadvantage to the students if they refuse. To avoid such situations, it is necessary to create a system to prevent such problems, such as setting up consultation services with specialists.

### Building trust through conversations

According to the results of this study, peer educators gradually gained the trust of their friends by approaching them and talking to them persistently, even if they were apprehensive at first. A relationship based on trust gradually formed through their continued discussions and learning from each other as well as through the students being able to voice their specific concerns to the peer educators. In a peer relationship, individuals recognize what they have in common, which contributes to a sense of camaraderie, and a desire to understand and support each other naturally arises [36], which was also true for the participants in this study. As adolescents are greatly influenced by the presence of peers [38], if both parties are in a trusting relationship, they may change their behavior. In Tanzanian school settings, it is common for teachers to use corporal punishment on students. However, the peer educators did not hit their friends, never got angry even in cases in which they were disrespected by the students, listened to what the other person had to say, and attempt to relate to their friends patiently and with a receptive attitude. It has been widely stated that a necessary quality for peer educators is not being emotional but being receptive and empathetic [33, 36]. The peer educators in this study interacted with their friends in this manner and built trusting relationships, and the students who observed the peer educators’ attitudes also built trusting relationships with other students in the same manner.

### Possibilities and challenges of peer-based adolescent education

This study suggests that discussing sex with peers, with whom the students might already have a trusting relationship, provides an opportunity for students to become more familiar with the societal/cultural issues related to sexual engagement. According to Prochaska and Velicer’s [39] Stages of Change Model, a person goes through a series of stages called indifference, interest, preparation, action, and maintenance before changing their behavior. As many secondary school students begin to develop an interest in sex or relationships around this time, it is important to provide them with the knowledge to make them aware of the necessity of behaving appropriately and to explain the consequences associated with engaging in sexual behavior. Furthermore, peer education on this topic might be more effective during this period when the students are indifferent about the topic and it is not yet relevant to them to allow them to freely express their thoughts in a safe space with their peers [40].

## Strengths and limitations

This study had unique strengths, such as eliciting the perceptions of adolescents from the rich data of 92 students. The study was conducted with the use of all authors’ strong language abilities in Swahili and English. Lincoln and Guba [41] stated that to establish scientific rigor of qualitative research, prolonged engagement is recommended. Although the data collection took place over a short period, the first author has lived in Tanzania for more than 3 years, understands the local language and culture, had visited the research site before this study, and plans to visit Tanzania in the future. In addition, it is a strength of the study that the data were triangulated by interviewing peer educators and students who were educated by peer educators.

This study also has several limitations. At the time of the FGD, there is a possibility that some of the students had difficulties in speaking in a group setting. Regarding the psychological and behavioral changes in students after adolescent education, it would be advisable to conduct in-depth interviews as well in the future to clarify what precisely is inhibiting or facilitating their activities as peer educators as well as personal feelings about peer education.

## Study implications for policy and research

As the situation surrounding adolescent pregnancy in Tanzania has recently been changing, it is timely to establish a comprehensive policy on adolescent education. By using the peer education approach, we may avoid the issue of increasing the number of teachers who teach this topic as they are in severe shortage. Instead, for peer education, teachers can act in a supervisory capacity and collaborate with NGO staff and medical professionals. Teachers can help peer educators schedule appointments to talk with students and support them in their activities, while medical professionals such as doctors and nurses can visit schools to provide accurate information when more specialized knowledge is required. As the issue is a private matter and varies among adolescents, adults need to create an environment that emphasizes cooperation and support for peer educators.

The results of this study have also raised new questions: Why were all of the students in this area able to engage in peer education activities with a desire to solve their problems, and why were some students positively accepted by peer educators when they received adolescent education, while others were not so pleasantly received? These issues could be topics for future research to help reduce the level of negative reactions received by the peer educators, including being insulted. In addition, to fully understand the culture and values of the community in the future, it is necessary to explore the perceptions of the students' parents and local professionals to better facilitate adolescent education. To this end, we believe that it is necessary to conduct individual interviews with students as well as school teachers, the students' parents, and community professionals with the aim of further examining peer-based adolescent education from a broader perspective.

## Conclusion

The students had both positive and negative perceptions of peer-based adolescent education. Both the peer educators and the students who received adolescent education felt that they gained confidence through the process based on the formation of a trusting relationship among them. We suggest that adult supervision and consultation would be beneficial for peer educators in cases in which they experience difficulties in implementing adolescent education.

## Supplementary Information

**Additional file 1.** The interview guide.

## Data Availability

The datasets used and analyzed during the current study are available from the corresponding author upon reasonable request.

## Abbreviation

FGD: Focus group discussion
