# Supplementary material for: Secondary school students’ and peer educators’ perceptions of adolescent education in rural Tanzania: a qualitative study
Source: Reprod Health. 2022 May 2;19:109. doi: 10.1186/s12978-022-01418-6 (PMC9063328; doi:10.1186/s12978-022-01418-6)
Supplement: Supplementary file 1 — Additional file 1. The interview guide. [file 12978_2022_1418_MOESM1_ESM.docx]

The interview guide

For peer educators:

(1) How do you feel about conducting adolescent education as a peer educator in schools?

Why do you think so?

For other students:

(2) How did you feel when you first received adolescent education from peer educators at school? How do you feel now that time has passed? Why do you think so?

For both:

(3) What are the problems related to sexuality in the environment in which you live?

(4) What do you feel you can do to address these issues as a secondary school student after conducting/receiving adolescent education?

For peer educators:

(5) Have you experienced any difficulties in conducting adolescent education through peer education? Why?

For other students:

(6) Are there any differences between receiving adolescent education from peer educators and from teachers, parents, or external NGO staff? Why?

(7) Do you feel that receiving adolescent education in school can increase interest in sex and lead to increased problematic behavior? Why do you think so?

For both:

(8) How do you feel about continuing adolescent education through peer education in the future? Why do you think so?
